# IL-1R/C3aR signaling regulates synaptic pruning in the prefrontal cortex of depression

**DOI:** 10.1186/s13578-022-00832-4

**Published:** 2022-06-17

**Authors:** Man-Man Zhang, Min-Xia Guo, Qiu-Ping Zhang, Xue-Qin Chen, Na-Zhi Li, Qing Liu, Jie Cheng, Shi-Le Wang, Guang-Hui Xu, Cheng-Fu Li, Ji-Xiao Zhu, Li-Tao Yi

**Affiliations:** 1 Department of Chemical and Pharmaceutical Engineering, College of Chemical Engineering, Huaqiao University, Xiamen, 361021 Fujian province People’s Republic of China; 2 Research Center of Natural Resources of Chinese Medicinal Materials and Ethnic Medicine, Jiangxi University of Chinese Medicine, Nanchang, 330004 Jiangxi province People’s Republic of China; 3 Xiamen Hospital of Traditional Chinese Medicine, Xiamen, 361009 Fujian province People’s Republic of China; 4 The First Affiliated Hospital of Xiamen University, Xiamen, 361003 Fujian province People’s Republic of China; 5 Xiamen Medicine Research Institute, Xiamen, 361008 Fujian province People’s Republic of China

**Keywords:** Complement C3, C3aR, Synaptic pruning, Palmitoylation, Depression

## Abstract

**Background:**

Major depressive disorder is characterized by not only monoamine neurotransmitters deficiencies but also persistent neuroinflammation. The complement system is an attractive therapeutic target for various inflammation-related diseases due to its early activation in inflammatory processes.

**Results:**

In the present study, the dynamic alteration of complement C3 and its receptor C3aR during the occurrence of depression and the mechanism of astrocyte-microglia IL-1R/C3/C3aR on synaptic pruning were investigated. The proteomic analysis firstly showed that chronic stress caused an elevation of C3. GO analysis indicated that complement system-mediated synaptic pruning signaling was involved in depression. The dynamic observation indicated that C3/C3aR was activated in the early onset and throughout the course of depression induced by lipopolysaccharide (LPS) and chronic stress. In contrast, C3aR blockade inhibited the hyperactivation of microglial APT2/DHHC7 palmitoylation cycle, which mediated the translocation of STAT3 and the expression of proinflammatory cytokines. Meanwhile, C3aR blockade also attenuated the synaptic pruning and enhanced the synaptogenesis in the prefrontal cortex of mice. Moreover, the blockade of IL-1R/NF-κB signaling pathway reduced the release of C3 from astrocyte.

**Conclusions:**

The current study demonstrates that astrocyte-microglia IL-1R/C3/C3aR activation causes the abnormal synaptic pruning in depression, and suggests that the activation of complement C3/C3aR may be particularly helpful in predicting the onset stage of depression.

**Supplementary Information:**

The online version contains supplementary material available at 10.1186/s13578-022-00832-4.

## Background

Depression is a common and severe mental disorder worldwide. The monoamine deficits hypothesis opens a window for elucidating the pathophysiology of depression and promoting the development of antidepressants. However, there are still 30% of depressed patients are resistant to antidepressants [1], and thus it is of great significance to explore the pathophysiology of depression from other biological and pathogenic factors.

Recently, increasing evidence has shown that neuroimmune is a key factor that correlates with major depression [2]. Complement system participates in both innate immunity and adaptive immunity. Because of its early and widespread reaction during immunity, accumulating evidence indicates that the complement system is involved in the pathophysiology of various diseases [3, 4]. Among the many complement molecules, C3 participates in not only the classical pathway but also the alternative and lectin pathways of the complement activation. This central position in complement activation makes C3 a putative target for the intervention of diseases [5–7]. Generally, C3 is expressed by astrocyte, and its receptor C3aR is expressed in neurons and notably in microglia [8, 9]. This astrocyte-microglia crosstalk through complement aggravates the release of C3 and the activation of microglia [10]. The involvement of neuroinflammation, such as microglial activation in the pathophysiology of depression, raises the possibility that complement activation may be an active participant in depression pathogenesis [11]. However, it remains unclear if complement C3/C3aR activation could be served as one of the distinguishing features in depression.

Synaptic pruning is widely accepted to be involved in the development and maturation of the brain [12]. Nevertheless, in case of neuroinflammation, the activation of microglia causes excessive synaptic pruning [13]. Microglia-induced synaptic pruning requires the guide of complements, especially complement C3 [14]. Generally, complement C3 binds the surface of pathogens or neuronal synapses, causing synaptic clearance by microglia. A recent study has shown that chronic stress induced an increase in C3aR expression and C3aR knockout mice displayed resilient features after chronic stress [15]. Another study found that the activation of C3a-C3aR signaling induced microglia polarization and neuroinflammation in depression [16]. However, the studies did not elucidate how the C3/C3aR signaling activation mediates the production of cytokines and the pathophysiology of depression. Besides, the mechanism of complement C3/C3aR in neuronal and synaptic damage of depression is not understood. Therefore, it requires a clearer understanding of the cellular response and molecular signals involved in complement system regulation. In the present study, we firstly evaluated the dynamic alteration of complement C3 and C3aR in the early onset and throughout the course of depression. We then investigated the underlying mechanism of C3/C3aR signaling pathway in synaptic pruning and palmitoylation cycle-mediated STAT3 activation in depression-like animals.

## Results

### C3aR blockade attenuated complement-mediated synaptic pruning by proteomic analysis

**Fig. 1:**
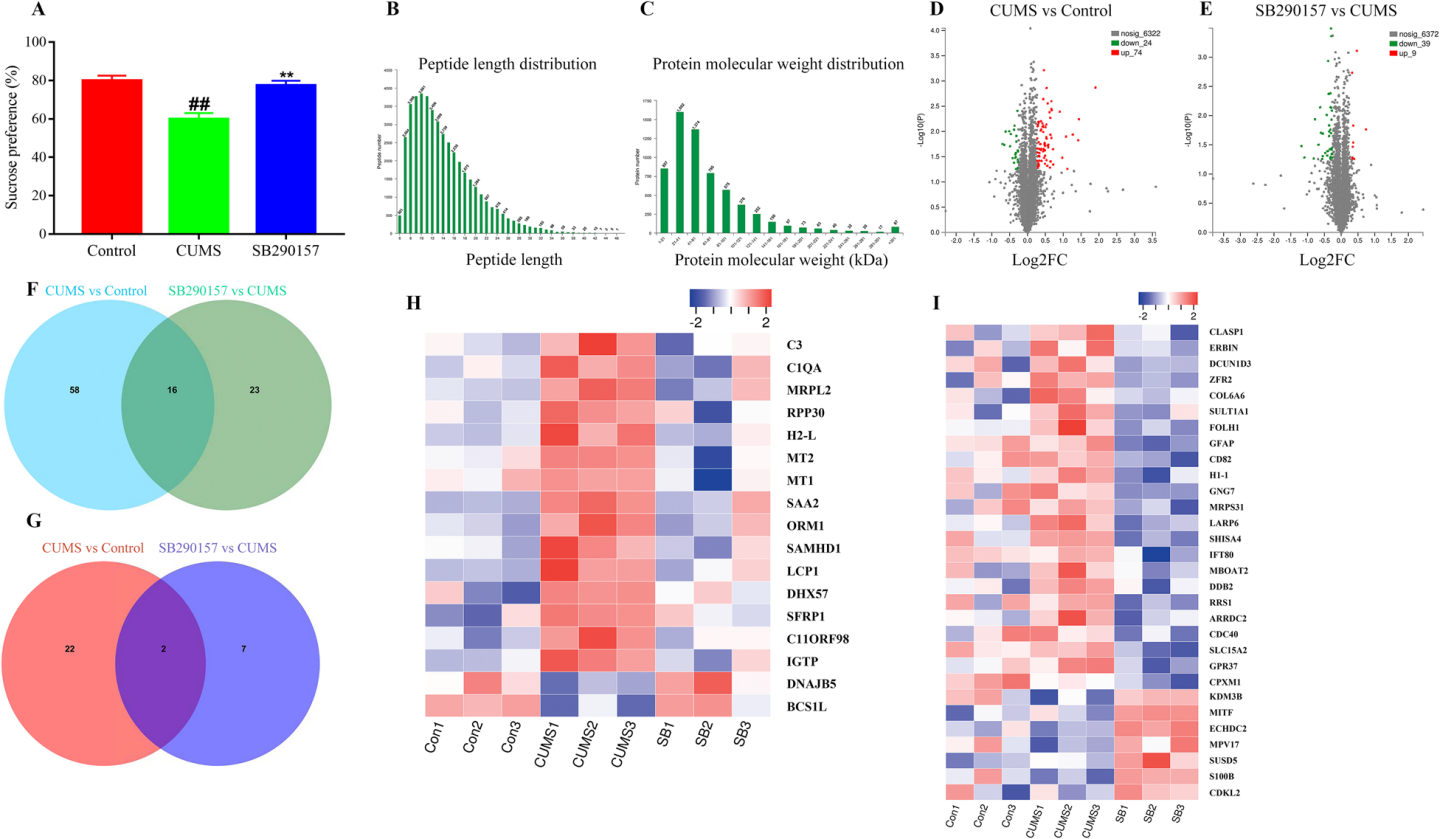
The proteomic analysis of C3aR blockade in the prefrontal cortex against depression. Sucrose preference (**A**). Peptide length distribution (**B**). Protein molecular distribution (**C**). Volcano plot of significant change proteins between Control and CUMS mice (**D**). Volcano plot of significant change proteins between CUMS and C3aR blockade mice (**E)**. Venn diagram of significant change proteins between Control and CUMS mice (**F**). Venn diagram of significant change proteins between CUMS and C3aR blockade mice (**G**). The heat map of the significant change proteins between Control and CUMS mice (**H**). The heat map of the significant change proteins between CUMS and C3aR blockade mice (**I**). Data from Panel A were obtained from 9 mice per group. Data from Panels **B**–**I** were obtained from 3 samples (three mice per sample) per group. ^##^*P* < 0.01 versus the Control group. ^**^P < 0.01 versus the CUMS group

To investigate the underlying signaling of complement system after C3aR blockade in depression, we performed TMT labeling-based quantitative proteomic analysis. The results from sucrose preference test (Fig. 1A) indicated that C3 signaling blockade reversed the depressive-like symptoms in mice exposed to chronic unpredictable mild stress (CUMS). The proteomic analysis firstly identified 6420 proteins (Fig. 1B, C). Compared with the control group, chronic stress induced 98 differentially expressed proteins (Fig. 1D). In detail, 74 proteins were over-expressed and 24 proteins were down-expressed. Compared with the CUMS mice, C3aR blockade induced 48 differentially expressed proteins (Fig. 1E). Among these proteins, 9 were upregulated and 39 were downregulated. Further analysis demonstrated that 16 proteins were significantly upregulated by CUMS but downregulated after C3aR blockade (Fig. 1F). Two proteins were markedly downregulated by CUMS but were upregulated after C3aR blockade (Fig. 1G). More importantly, complement C3 was significantly increased by chronic stress, while C3aR blockade decreased the levels of C3 (Fig. 1H, I). Besides, C1q, another factor of complement system, was also inhibited by C3aR blockade compared with CUMS group.

**Fig. 2:**
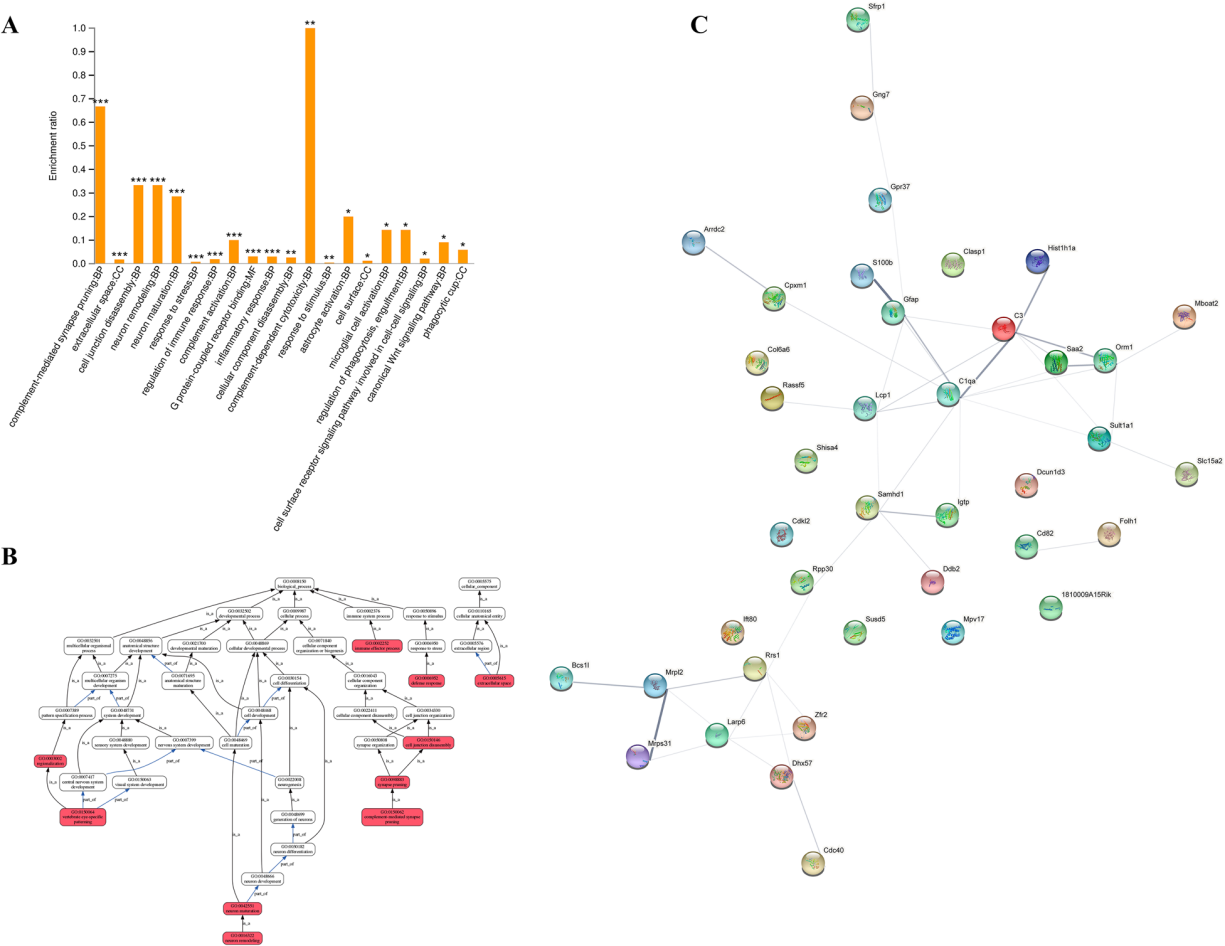
The functional analysis of proteomic in depression. The histogram of GO enrichment analysis shows the proteins between CUMS/Control and C3aR blockade/CUMS (**A**). The biological process between CUMS/Control and C3aR blockade/CUMS (**B**). The protein interaction network analysis between CUMS/Control and C3aR blockade/CUMS (**C**). ^*^P < 0.05, ^**^P < 0.01 and ^***^P < 0.01. Data were obtained from 3 samples (three mice per sample) per group

Gene Ontology (GO) analysis of the changed proteins in the prefrontal cortex between Control/CUMS and CUMS/C3aR blockade demonstrated a significant regulation of complement-mediated synapse pruning, cell surface receptor signaling pathway involved in cell–cell signaling, phagocytosis, microglial activation, and astrocyte activation (Fig. 2A). As shown in Fig. 2B, C3aR blockade could regulate complement blockade could regulate complement-mediated synapse pruning-cell junction disassembly-cell junction/component organization-cellular component biogenesis pathway. Then, we further performed protein interaction network analysis to elucidate the possible interaction of differential expressed proteins (Fig. 2C). The results indicated that C3aR blockade caused the down-expression of C3, which regulated SAA2-ORM, SULT1A1-SLC15A2 signaling.

### LPS caused C3/C3aR upregulation prior to depressive-like symptoms

**Fig. 3:**
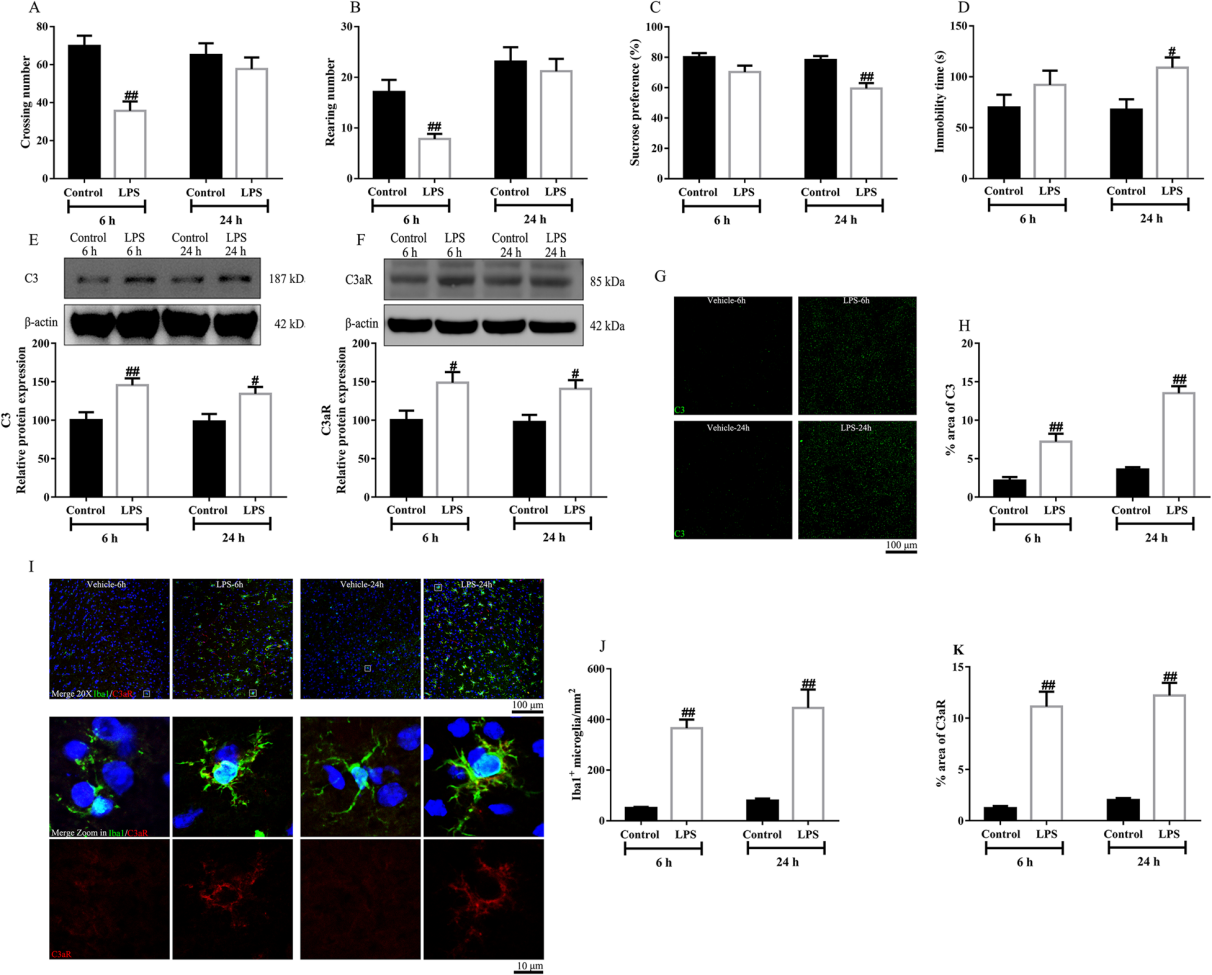
LPS caused the elevation of C3 and its receptor C3aR in the prefrontal cortex after 6 h and 24 h. The effects of LPS on crossing number (**A**), rearing number (**B**), sucrose preference (**C**) and immobility time (**D**) in mice. LPS enhanced C3 (**E**) and C3aR (**F**) expression. Representative photographs of C3(green) (**G**) and the statistical results (**H**). Representative photographs of Iba1(green)/C3aR(red)/DAPI(blue) (**I**). LPS increased microglial activation (**J**) and C3aR (**K**) expression. Data were obtained from 5–10 samples per group. ^#^*P* < 0.05 and ^##^*P* < 0.01 versus the Control-vehicle group

LPS-treated mice exhibited sickness at 6 h after injection as the locomotor activity was decreased (Fig. 3A, B). The mice were not induced to depression at this time point as the sucrose preference between 0 and 6 h was not changed. Consistently, the immobility time in LPS-treated mice was not increased at 6 h after LPS treatment. On the contrary, LPS induced depressive-like behaviors but not sickness symptoms at 24 h post injection. These observations indicated that only sickness symptoms but not depressive-like symptoms occurred at 6 h post LPS injection (Fig. 3C, D), while only depressive-like symptoms but not sickness symptoms appeared at 24 h post LPS injection. These results were consistent with a previous publication showing depressive-like behaviors occurred at 24 h post LPS injection at which the sickness symptoms disappeared [17].

C3, the most abundant complement protein, plays a crucial function in the immunological cascades. C3/C3aR levels were detected at the pre-onset (6 h post LPS injection) and onset (24 h post LPS injection) of depression. C3 levels in the prefrontal cortex were significantly increased at both 6 h and 24 h after LPS treatment. In addition, the C3aR levels were increased both at the pre-onset and onset stage (Fig. 3E, F). A previous study using single-cell transcriptomic profiling showed that C3aR was cell-type-specifically expressed, dominantly in microglia and less in neurons [9]. Using RNA in situ hybridization, another study indicated that microglia possessed high levels of C3aR, and the effect of C3 was absent in C3aR null microglia. In the present study, we also checked the location of C3aR in brain by immunofluorescence with 3D reconstruction. The results showed that C3aR is expressed predominantly in microglia, lowly in neurons, but not in astrocytes (Additional file 1: Fig. S1). In this respect, we only assessed C3aR in microglia in the following measurements. In parallel to the change of C3/C3aR levels in western blot, the density of C3, the number of microglia or the density of C3aR (Fig. 3G–K) was increased at both 6 h and 24 h post LPS injection.

### Chronic stress induces C3/C3aR in the early onset of depression

**Fig. 4:**
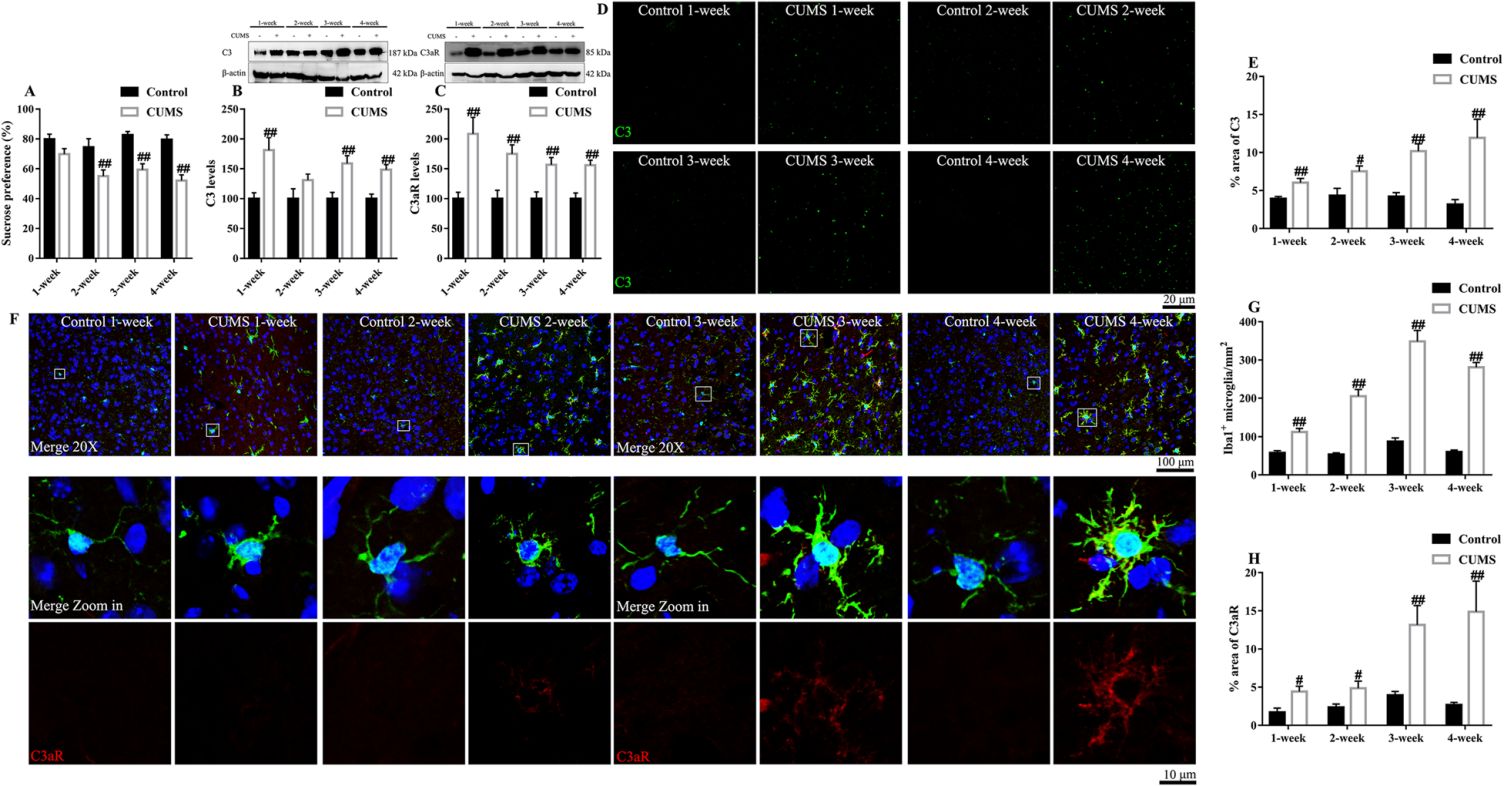
The dynamic change of C3/C3aR in the prefrontal cortex in a 4-week chronic stress procedure. The dynamic change of sucrose preference (**A**), C3 (**B**) and C3aR (**C**) levels in mice. Representative photographs of C3(green) (**D**) and the statistical results (**E**). Representative photographs of Iba1(green)/C3aR(red)/DAPI(blue) (**F**). The dynamic change of microglial activation (**G**) and C3aR expression (**H**). Data were obtained from 5–10 samples per group. ^#^*P* < 0.05 and ^##^*P* < 0.01 versus the Control-vehicle group

To verify if the activation of C3 signaling is a pre-onset index of depression, we assessed C3/C3aR in mice during the induction of depression and at the onset of depression. We thus performed sucrose preference test and detected C3/C3aR levels every week in mice exposed to CUMS (Fig. 4), a widely used depression model which mimics major depression in humans. The results showed that C3/C3aR levels were upregulated among the whole procedure of CUMS; however, anhedonia behavior occurred two weeks later after CUMS. These findings indicate that the expression of C3/C3aR increases during the development of depression, and that complement C3 signaling activation might be a pre-onset index of depression.

### C3/C3aR signaling blockade alleviated depressive-like behaviors, decreased C3/C3aR levels in chronic stress-induced depression

**Fig. 5:**
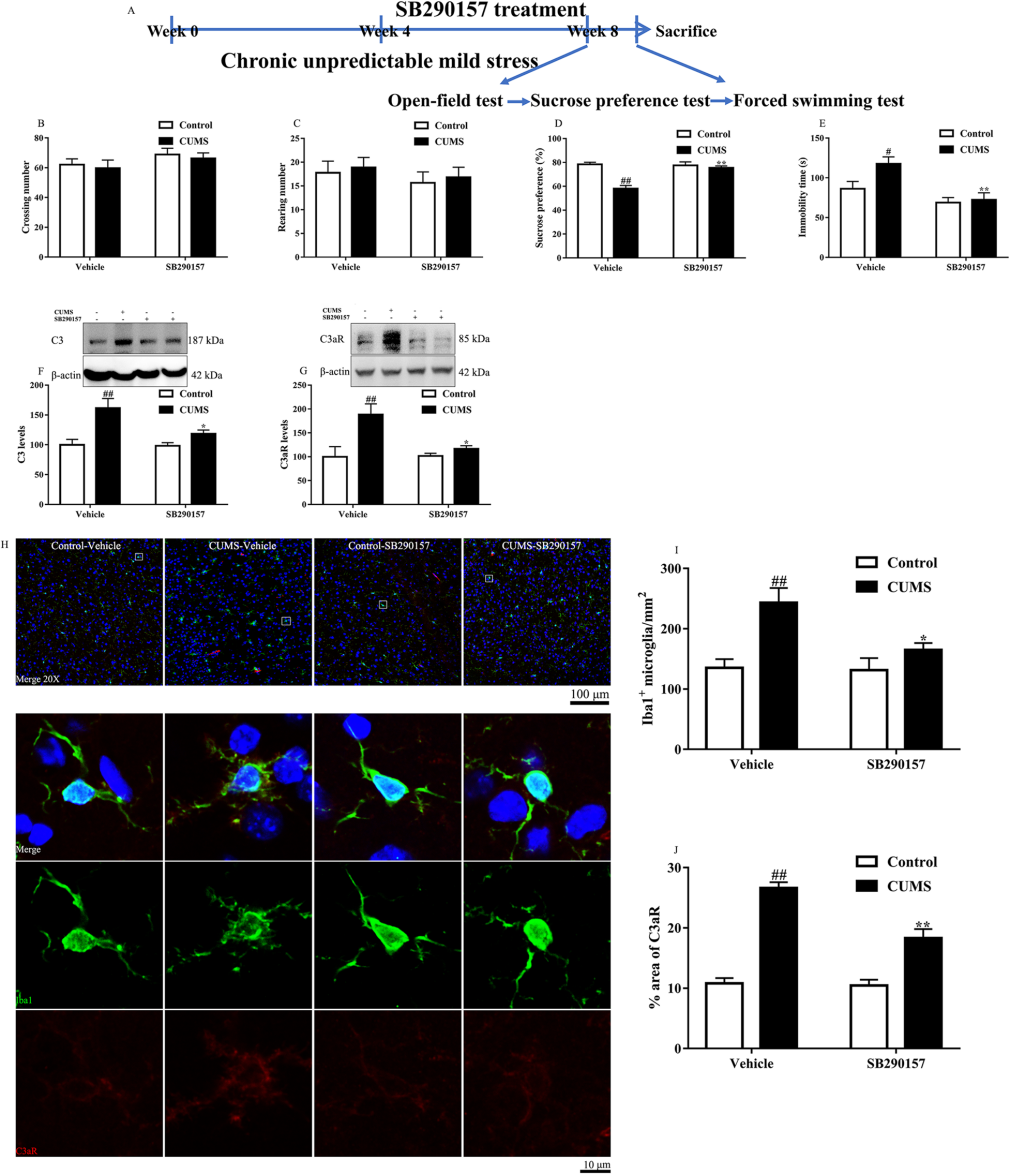
The timeline of the C3aR blockade experiment (**A**). The effects of C3aR blockade by SB290157 on crossing number (**B**), rearing number (**C**), sucrose preference (D) and immobility time (**E**) in mice C3aR blockade reduced the levels of C3 (**F**) and C3aR (**G**). Representative photographs of Iba1(green)/C3aR(red)/DAPI(blue) (**H**). C3aR blockade inhibited microglial activation (I) and C3aR (**J**) expression. Data were obtained from 5–16 samples per group. ^#^*P* < 0.05 and ^##^*P* < 0.01 versus the Control-vehicle group. ^*^P < 0.05 and ^**^P < 0.01 versus the CUMS-vehicle group

Then the effects of C3aR blockade with SB290157 were investigated in the present study (Fig. 5A). Firstly, neither C3aR blockade nor CUMS altered the locomotor activity (Fig. 5B, C). However, C3aR blockade alleviated the depressive-like behaviors such as anhedonia and despair in response to CUMS procedure (Fig. 5D, E). In addition, CUMS caused an elevation of C3 and C3aR in the prefrontal cortex, while C3aR blockade attenuated the levels (Fig. 5F, G). Furthermore, we used immunofluorescence to assess the number of microglia and the expression of C3aR within microglia in the prefrontal cortex. The results indicated that C3aR blockade decreased the number of Iba1 labeled microglia and inhibited the expression of C3aR within microglia in CUMS mice (Fig. 5H–J).

### C3/C3aR signaling blockade inhibited microglial APT2/DHHC7 mediated STAT3 translocation and proinflammatory cytokine expression in the prefrontal cortex

**Fig. 6:**
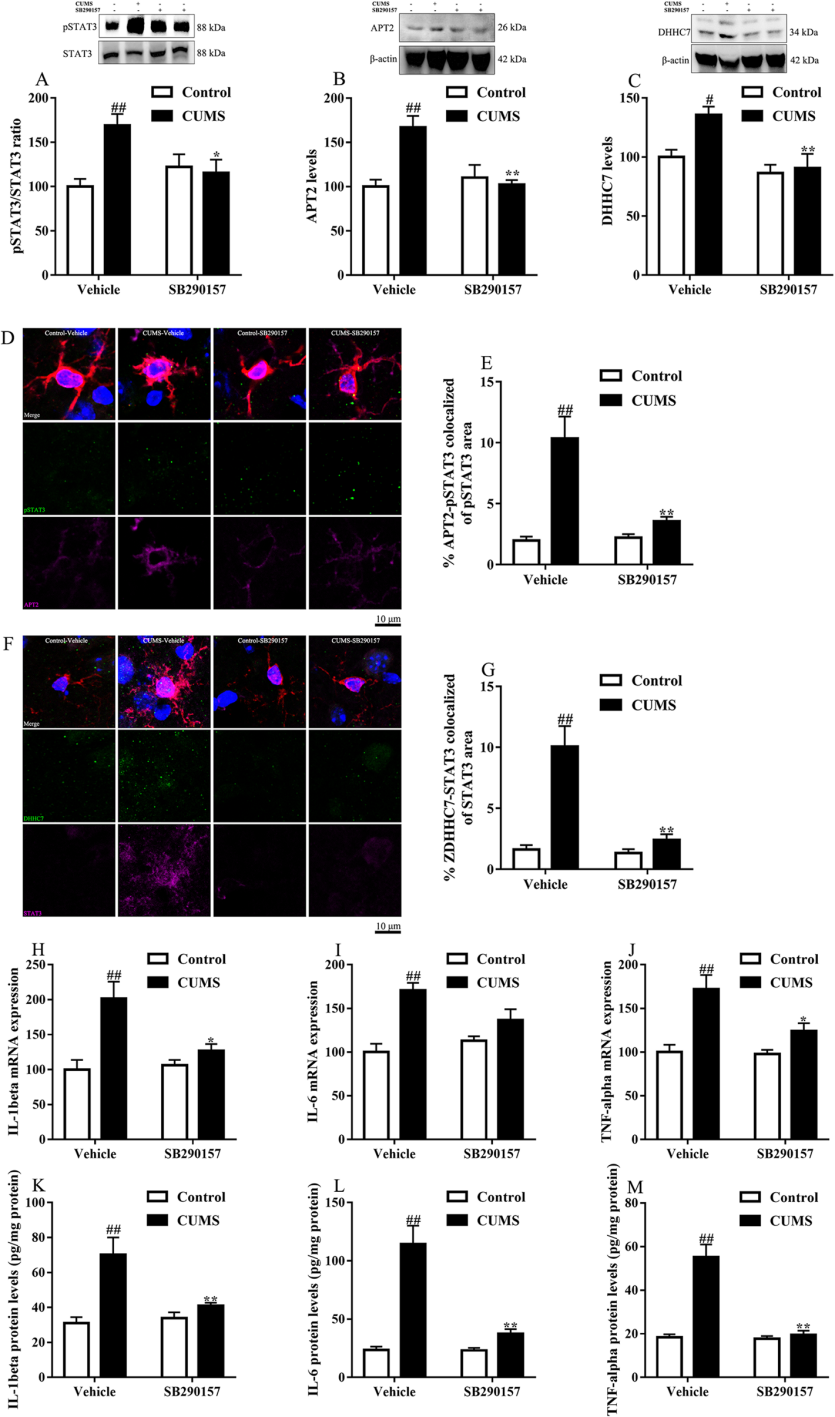
C3aR blockade inhibited APT2/DHHC7 palmitoylation-mediated STAT3 phosphorylation in the prefrontal cortex. The levels of APT2 (**A**), DHHC7 (**B**) and pSTAT3 (**C**) were decreased after C3aR blockade. Representative photographs (**D**) of Iba1(red)/pSTAT3(green)/APT2(purple)/DAPI(blue) and the co-localized ratio (**E**) of pSTAT3 and APT2. Representative photographs (**F**) of Iba1(red)/DHHC7(green)/STAT3(purple)/DAPI(blue) and the co-localized ratio (**G**) of STAT3 and DHHC7. C3aR blockade decreased proinflammatory cytokines both in mRNA expression (**H**–**J**) and protein levels (**K**–**M**). Data were obtained from 5–6 samples per group. ^#^*P* < 0.05 and ^##^*P* < 0.01 versus the Control-vehicle group. ^*^P < 0.05 and ^**^P < 0.01 versus the CUMS-vehicle group

The transcription factor STAT3 has been recently verified to be recruited to cell membrane and phosphorylated under C3aR activation [18]. The phosphorylated STAT3 is translocated to the nucleus and thus activates its target genes. Likewise, in the chronic stress-induced depression mice, the pSTAT3/STAT3 ratio increased in the prefrontal cortex. C3aR blockade reversed the increase of pSTAT3/STAT3 ratio in the prefrontal cortex (Fig. 6A), which was partly consistent with the fact that inhibition of microglial STAT3 alleviates depressive-like behaviors [19].

A recent study demonstrated that palmitoylation promoted the translocation of STAT3 to the membrane and nucleus [20]. DHHC7 palmitoylated STAT3 and facilitated its membrane recruitment and the following phosphorylation by its upstream activator. Then, APT2 depalmitoylated pSTAT3 and facilitated its nucleus translocation. This seemingly futile palmitoylation-depalmitoylation cycle ensures that most phosphorylated STAT3 is present in the cell. We assessed DHHC7 and APT2 protein for the first time in depression-like animals. The western blot analysis indicated that DHHC7 and APT2 levels were increased in response to chronic stress (Fig. 6B, C). In contrast, C3aR blockade decreased DHHC7 and APT2 levels. The same trend was observed in the change of pSTAT3/STAT3 levels.

Considering that APT2 mainly acts with pSTAT3 over STAT3 and DHHC7 acts with both states of STAT3, we performed immunofluorescence to colocalized APT2/pSTAT3 and DHHC7/STAT3. The results showed that APT2/DHHC7 mediated palmitoylation-depalmitoylation cycle was upregulated in mice exposed to chronic stress, indicating the membrane and nucleus translocation of STAT3. In contrast, C3aR blockade significantly attenuated the highly colocalized area of APT2/pSTAT3 and DHHC7/STAT3 in mice exposed to chronic stress (Fig. 6D–G).

We next evaluated if the target genes of STAT3 were changed during the occurrence of depression and C3aR blockade (Fig. 6H–M). The results indicated a significant increase in mRNA and protein levels of proinflammatory cytokines in chronic stress-induced mice, while C3aR blockade reduced the expression.

### C3/C3aR signaling blockade decreased synapse localized C3 levels and restored synaptic engulfment in depression

**Fig. 7:**
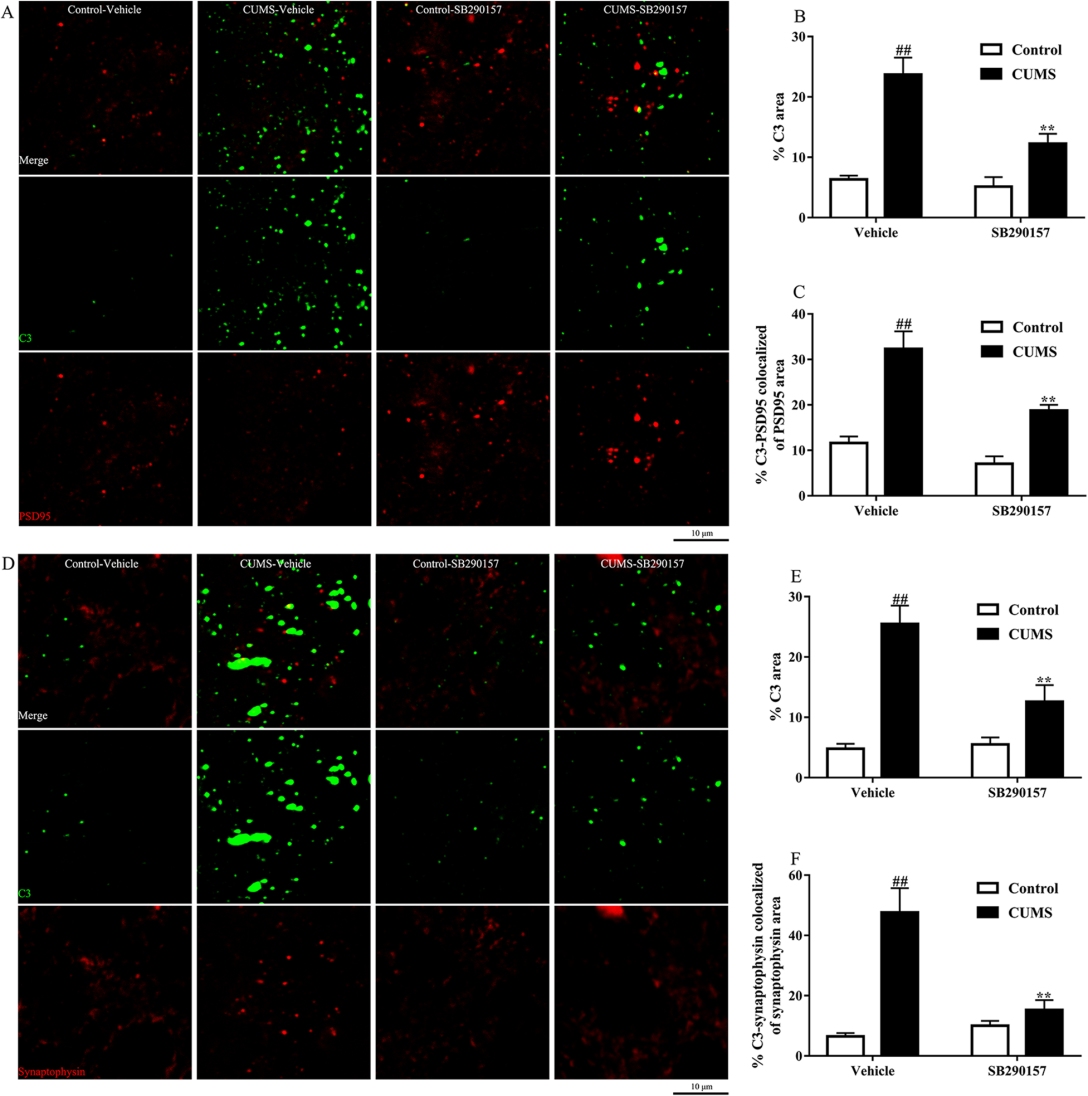
C3aR blockade prevented C3 tagging with synaptic proteins in the prefrontal cortex of CUMS mice. Representative photographs of PSD95(red)/C3(green)/DAPI(blue) (**A**). C3aR blockade decreased the ratio of C3 area (**B**) and the ratio of PSD95 colocalized with C3 (**C**). Representative photographs of Synaptophysin(red)/C3(green)/DAPI(blue) (**D**). C3aR blockade decreased the ratio of C3 area (**E**) and the ratio of synaptophysin colocalized with C3 (**F**). Data were obtained from 5 samples per group. ^##^*P* < 0.01 versus the Control-vehicle group. ^**^P < 0.01 versus the CUMS-vehicle group

Microglia play a dual role in the brain. They act protectively to neurons in a quiescent state. However, once the microglia are activated, they will act with an opposite effect on neurons, such as excessive synapse pruning. Typically, the engulfment and elimination of synapses are mediated by complement C3 [14]. To provide direct evidence that synapse-localized C3 induces microglia-mediated synapse engulfment and elimination, we stained C3 with postsynaptic protein PSD95 (Fig. 7A–C) and presynaptic protein synaptophysin (Fig. 7D–F). The results showed that C3 was highly enriched at postsynaptic and presynaptic terminals in mice induced by CUMS. On the contrary, mice with C3aR blockade showed less synapse-localized C3 levels.

**Fig. 8:**
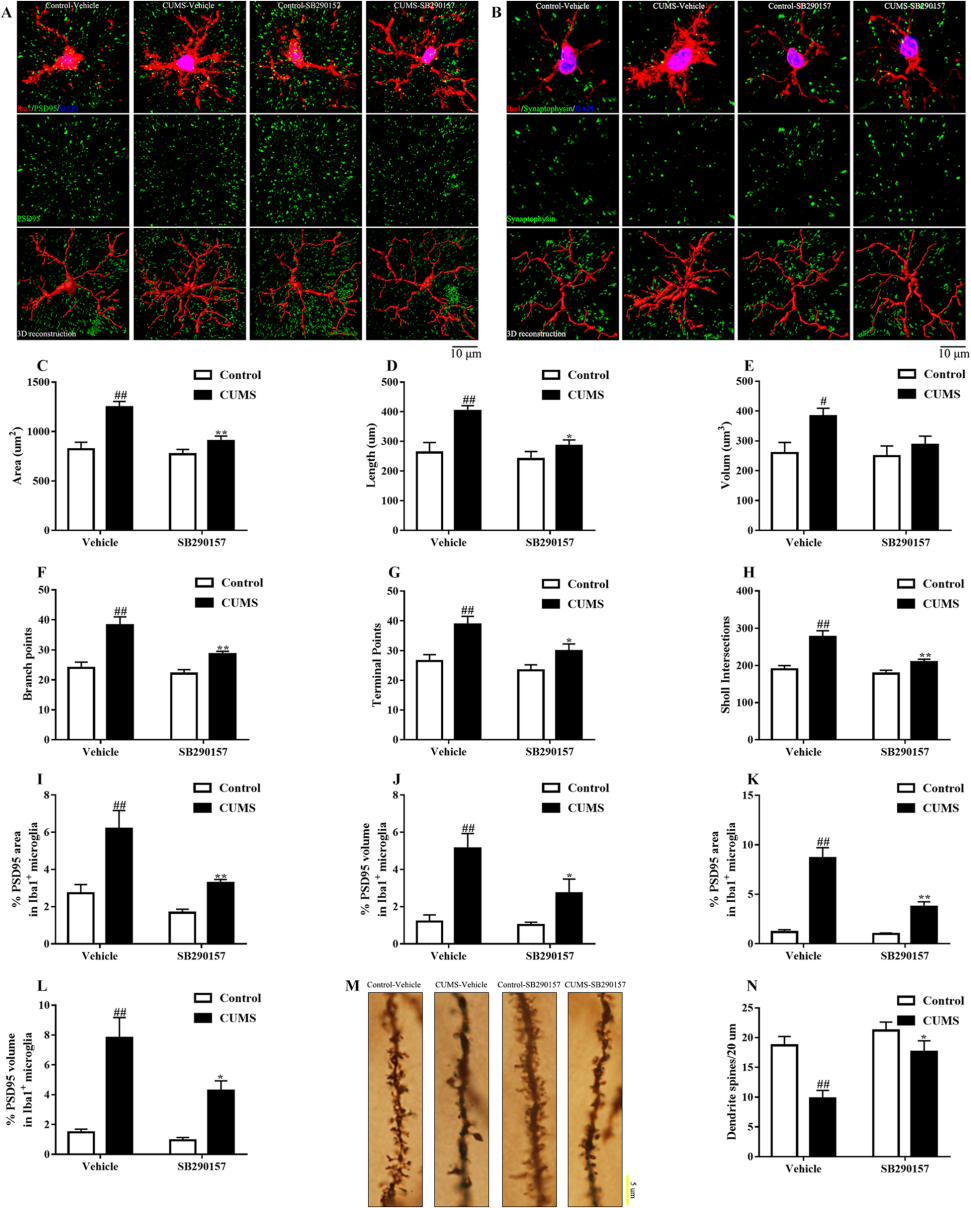
C3aR blockade inhibited synaptic pruning in the prefrontal cortex of CUMS mice. Representative photographs of Iba1(red)/PSD95(green)/DAPI(blue) and the 3D reconstruction (**A**). Representative photographs of Iba1(red)/synaptophysin (green)/DAPI(blue) and the 3D reconstruction (**B**). C3aR blockade restored the microglial morphology (**C**-**H**), prevented the engulfment of PSD95 by microglia according to area (**I**) and volume (**J**) analysis, and prevented the engulfment of synaptophysin by microglia according to area (**K**) and volume (**L**) analysis. Representative photographs of dendritic spines by Golgi staining (**M**). C3aR blockade enhanced dendritic spines in CUMS-induced mice (**N**). Data were obtained from 5 samples per group. ^##^*P* < 0.01 versus the Control-vehicle group. ^*^P < 0.05 and ^**^P < 0.01 versus the CUMS-vehicle group

To further examine whether synapse-localized C3 contributed to synapse loss in depression, we stained microglia (Iba1), postsynapse (PSD95), or presynapse (synaptophysin) in the prefrontal cortex (Fig. 8A–H). In agreement with the data from synapse localized C3 levels, the engulfed area and volume of PSD95 and synaptophysin were decreased in C3aR blockade mice (Fig. 8I–L). The microglial morphology was also restored by C3aR blockade. Moreover, Golgi staining indicated that C3aR blockade enhanced the dendritic spines in response to chronic stress (Fig. 8M, N). These data support a complement-mediated therapy by decreasing microglia-induced engulfment and synapses elimination.

### C3 release was inhibited by antagonizing IL-1R/NF-κB/C3 signaling in astrocyte

**Fig. 9:**
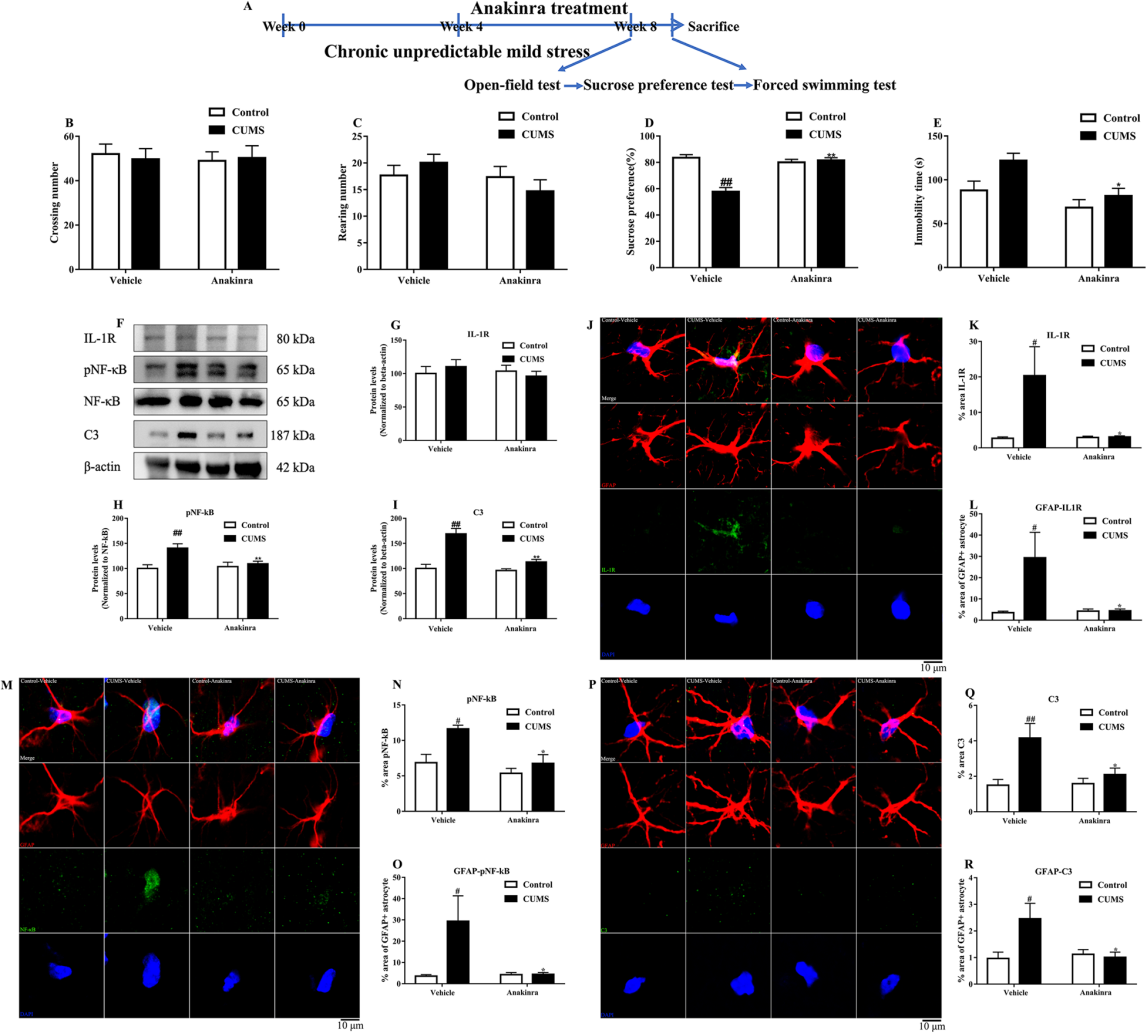
IL-1R blockade inhibited astrocyte IL-1R/NF-κB/C3 signaling pathway in the prefrontal cortex of CUMS mice. The timeline of the IL-1R blockade experiment (**A**). The effects of IL-1R blockade by Anakinra on crossing number (**B**), rearing number (**C**), sucrose preference (**D**) and immobility time (**E**) in mice. Representative photographs of bands in western blot (**F**). IL-1R blockade did not decrease IL-1R (**G**), but decreased pNF-κB/NF-κB (**H**) and C3 (**I**) levels in the prefrontal cortex. Representative photographs of GFAP positive cells (red), IL-1R (green) and DAPI labeled nuclear (blue) (**J**). IL-1R blockade prevented the increase of IL-1R in the prefrontal cortex (**K**) and astrocyte (**L**). Representative photographs of GFAP positive cells (red), pNF-κB (green) and DAPI labeled nuclear (blue) (**M**). IL-1R blockade prevented the increase of pNF-κB in the prefrontal cortex (N) and astrocyte (**O**). Representative photographs of GFAP positive cells (red), C3 (green) and DAPI labeled nuclear (blue) (**P**). IL-1R blockade prevented the increase of C3 in the prefrontal cortex (**Q**) and astrocyte (**R**). Data were obtained from 5–20 samples per group. ^#^*P* < 0.05 and ^##^*P* < 0.01 versus the Control-vehicle group. ^*^P < 0.05 and ^**^P < 0.01 versus the CUMS-vehicle group

To assess whether cytokine signaling blockade could attenuate C3 release and the behavioral deficits in depression-like animals, Anakinra, an IL-1R antagonist was administered daily for 4 consecutive weeks in mice exposed to CUMS. The experimental timeline was shown in Fig. 9A. As shown in Fig. 9B, C, neither crossing number nor rearing number was changed by IL-1R blockade. Additionally, IL-1R blockade increased sucrose preference and decreased immobility time in mice exposed to CUMS (Fig. 9D, ). Then the activation of IL-1R dependent C3 signaling pathway was evaluated. We evaluated the total IL-1R, pNF-κB/NF-κB and C3 levels in the prefrontal cortex (Fig. 9F–I). The data from Western blot showed that CUMS induced an elevation of pNF-κB/NF-κB ratio and C3 levels, which were reversed by IL-1R blockade. C3, the most commonly used specific marker for A1 astrocytes [21], was significantly upregulated in astrocyte-specific IκBα (inhibitor kappa B alpha, an inhibitor of NFκB) deletion mice and as a direct target of NFκB in astrocytes [22]. Thus, we tried to assess whether IL-1R blockade could mediate the release of complement C3 from astrocytes. Immunostaining was used to detect the levels of IL-1R (Fig. 9J–L), pNF-κB (Fig. 9M–O) and C3 (Fig. 9P–R) within astrocyte. The results from immunostaining showed a dramatic increase of IL-1R/pNF-κB/C3 intensity in GFAP-positive astrocytes in CUMS mice that was almost completely prevented by IL-1R blockade.

## Discussion

To the best of our knowledge, our present study provides direct evidence to support that complement C3/C3aR is activated in the early onset and during the induction of depression in response to LPS injection and chronic stress. Our study also demonstrates that C3aR-APT2/DHHC7 palmitoylation-mediated STAT3 activation and synaptic pruning are involved in the pathophysiology of depression.

The TMT quantitative proteomic analysis revealed a significant alteration of proteins related to the complement-mediated inflammatory pathway in chronic stress-induced animals. Strikingly, C3aR blockade normalized the protein expression of more than half of the upregulated genes (39 out of 74 proteins) and reversed the astrocyte activation and microglial activation. These proteins are mainly involved in the complement-mediated synapse pruning. Therefore, we suppose that blockade of C3aR exerts its primary effect by targeting the microglial protein expression.

The observation above provided a possibility that C3 participates in the pathophysiology of depression. The clinical investigation also found the increased levels of C3 in the serum and prefrontal cortex of patients with major depression, and these levels were reduced with the intervention of antidepressants [15, 23]. To further elucidate the role of C3 and its receptor C3aR, we next focused on the change of C3/C3aR in different depression-like animals. A previous study showed that central C3 was induced in response to LPS injection [24]. We firstly focused on the C3 and C3aR expression after different time points after LPS injection. The results showed that C3 and C3aR levels were typically elevated at 6 h (sickness) and 24 h (depression) post LPS injection. Given that sickness is a form of depression [25], this results suggest that C3/C3aR signaling is typically activated at pre-onset and onset stages of depression.

Next, the dynamic evaluation of C3/C3aR expression was also investigated in mice induced by chronic stress. The C3/C3aR expression was activated at 1 week post chronic stress, while depression-like symptoms appeared at 2 weeks post chronic stress. In this respect, the intervention of C3aR signaling might prevent the occurrence of the disease. A recent study demonstrated that C3aR inactivation restored the neuroinflammatory action in the brain, which implies that C3aR regulates inflammation-related transcription factors and their targets cytokines [18]. According to a previous study [26], microglia are the primary native cell type expressing C3aR in the brain, although it can also be observed in neurons according to a previous study and our immunofluorescence data [10]. Therefore, the present study focused on the C3aR and its signaling pathway, mainly in microglia. STAT is one of the transcriptional factors mediated by inflammation and immunity [27]. A recent study using transcription factor network analysis revealed that STATs family, including STAT1, STAT3, STAT5a, and STAT5b, increased in memory deficit mice. However, only STAT3 expression was reversed by C3aR knockout in microglia [18]. These results were consistent with the independent proteomics analysis by the RPPA platform [18]. In addition, there is evidence that microglial STAT3 activation induced neuronal cell death by regulating neurite outgrowth, neurotransmitter receptors, and synapse-related proteins [28, 29]. Taken together, the present study mainly measured STAT3-related signaling in microglia. The results showed that pSTAT3 levels were upregulated by CUMS, while C3aR antagonist decreased the levels. In this respect, the observations above suggest that microglial C3aR-STAT3 inactivation mediates the antidepressant-like effects in CUMS mice. Consistently, a previous study also showed that microglia-specific STAT3 knockout mice produced antidepressant-like effects in mice as the depressive-like behaviors were fully improved in several behavioral tests [19].

In addition to phosphorylation-dephosphorylation, palmitoylation-depalmitoylation, another reversible posttranslational modification, also regulates the function of numerous proteins in the brain [30]. DHHC7, a key conserved palmitoyl acyltransferase, regulates synaptic transmission and synaptic plasticity in the brain [31]. DHHC7 is commonly suggested to be crucial for non-genomic rapid responses to steroid hormones to brain organization [32]. Besides, palmitoylation is involved in microglia proliferation and neuroinflammation [33]. A recent study showed that a palmitoylation cycle modulated the activation and nucleus translocation of STAT3 in response to inflammation [20]. The palmitoylation cycle is regulated by DHHC7 and APT2. Briefly, DHHC7 palmitoylates STAT3 and promotes its membrane recruitment and phosphorylation to STAT3. While APT2 facilitates pSTAT3 nuclear translocation by pSTAT3 depalmitoylation. The present study showed that DHHC7 and APT2 were significantly elevated by CUMS, while C3aR blockade inhibited the expression of DHHC7 and APT2 in the prefrontal cortex. Besides, the immunofluorescence detected the high co-localization of APT2-pSTAT3 and DHHC7-STAT3 in response to chronic stress, indicating that the palmitoylation cycle induces STAT3 signaling in depression. C3aR blockade inhibited APT2/DHHC7 mediated palmitoylation cycle and further inactivated STAT3 phosphorylation as well as proinflammatory cytokine expression. To the best of our knowledge, our study for the first time demonstrated that the palmitoylation cycle-mediated STAT3 phosphorylation was activated in depression but antagonized by C3aR signaling silence.

The complement system mediates synaptic pruning to optimize neuronal connections in the central nervous system [34]. Typically, classical complement cascade protein C3 is localized at developing synapses and mediates synaptic pruning by microglia [35]. In the present study, CUMS caused the upregulation of co-localization of C3 with PSD95 and synaptophysin, indicating that excessive neurons were tagged by the neuroinflammatory system. On the contrary, C3aR blockade decreased the co-localization of C3 with synaptic proteins, suggesting the reduction of tagged synapses. In the immune system, synapses tagged by immune molecules will be eliminated by microglia that express complement receptors [36]. C3 binds with C3aR to regulate cytokine responses, synaptic formation, and microglial pruning [37]. In this system, complement C3 combines with C3aR to locate and mark synapses that need pruning [38]. Microglia prunes excess synapses when it is inappropriately activated [39]. The present study also showed that the microglia induced by chronic stress displayed multi-branched ramification, which like an amoeboid shape, is partly consistent with a previous study [40]. Consistently, synapse engulfment was enhanced by microglia in CUMS mice as increased synaptic proteins were within the body of Iba1-labeled microglia. In parallel to C3/PSD95 and C3/synaptophysin co-localization changes, C3aR blockade decreased the engulfment of synaptophysin/PSD95 in the body of microglia. Under normal state, C3 can bind all synapses, but only the weaker synapses are labeled by C3 and pruned by microglia [41]. These observations indicated that excessive normal synapses were tagged as the weaker synapses to be eliminated by microglia during CUMS-induced depression, which might be involved in the pathophysiology of depression.

Cytokines were shown to regulate the release of C3 in the central nervous system [42, 43]. In this respect, we imaged that IL-1β blockade could regulate the release of C3. IL-1 signaling pathway plays an essential role in the pathogenesis of inflammation-mediated mental diseases such as depression [44]. IL-1R is the receptor for IL‐1β, thereby mediating IL-1-dependent activation. Generally, IL-1R is expressed at low abundance but with a very high affinity in different cell types such as astrocytes, endothelial cells, and Müller glia. By constructing IL-1Rr/r mice (IL-1R-null) and GFAPCre-IL-1Rr/r mice (IL-1R expressed only in astrocytes), researchers found that the effects of IL-1β were mediated by signaling through IL-1R expressed in astrocytes [45]. Therefore, together with the report that C3 is the most commonly used specific marker for A1 astrocytes [21], we tried to elucidate the effects of IL-1R antagonist in the astrocyte. NF-κB has been verified as the downstream effector of IL-1R signaling [46]. In addition, NF-κB is a transcription factor, and the complement C3 promoter contains two κB binding sites [22, 47]. In this context, IL-1R/NF-κB/C3 signaling pathway was evaluated in the present study. Our study found that the IL-1R blockade alleviated astrocyte IL-1R levels, NF-κB phosphorylation and C3 levels induced by CUMS, demonstrating that astrocyte IL-1R/NF-κB/C3 mediates the pathophysiology of depression. Considering that IL‐1β is mainly expressed in microglia, the blockade of C3aR could totally inhibit both the release of C3 and the activation of its downstream signaling.

## Conclusions

**Fig. 10:**
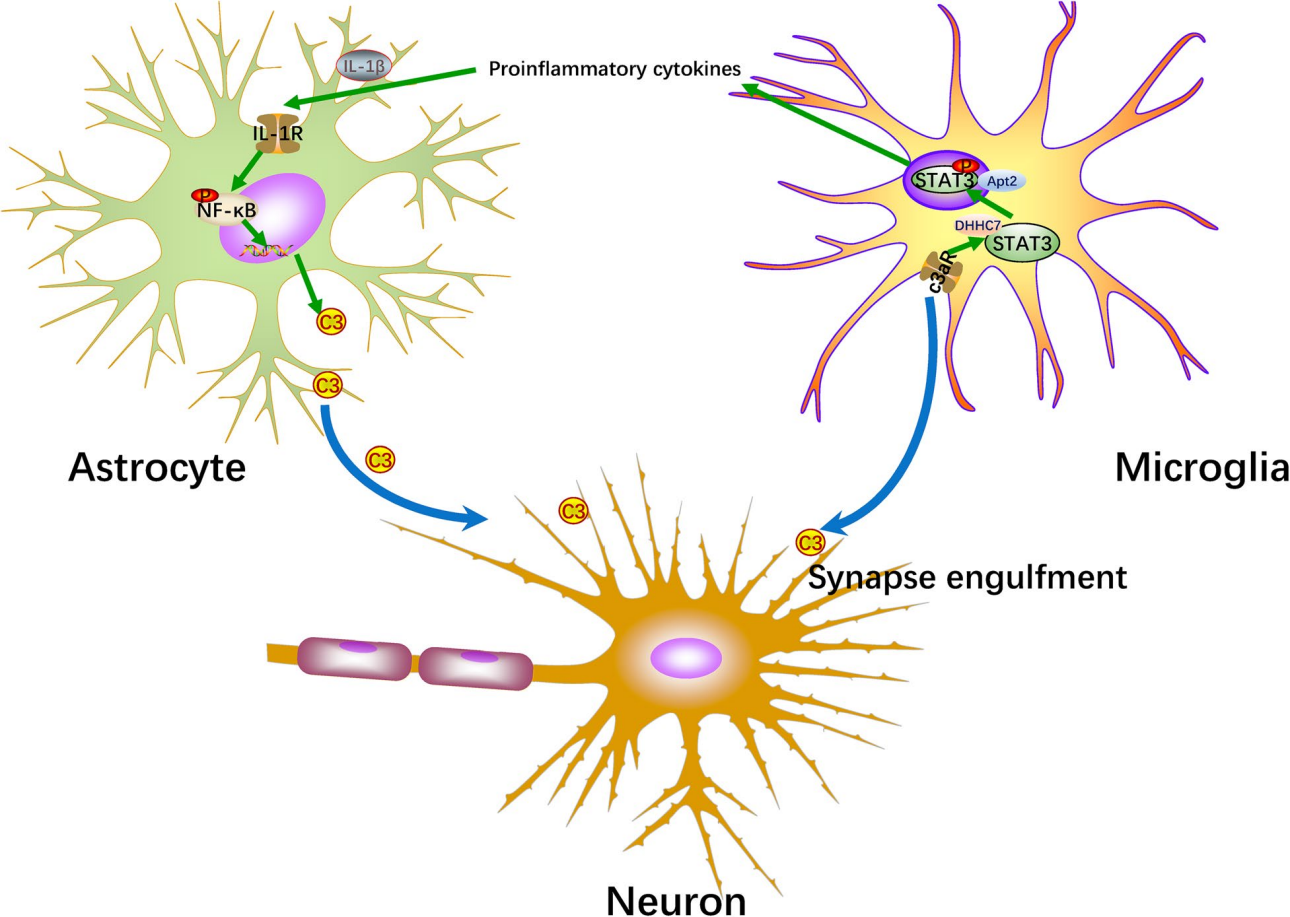
Schematic diagram shows that the activation of astrocyte IL-1R/NF-κB/C3 signaling and microglial C3aR/APT2/DHHC7 palmitoylation mediates the pathophysiology of depression. Complement C3 is over-expressed by astrocyte IL-1R/NF-κB/C3 pathway in response to stress. Astrocyte-neuron crosstalk mediates through the release of complement C3 from astrocyte and the localization of C3 with neurons. Astrocyte-microglia crosstalk mediates through C3-C3aR chemotaxis. Microglia-neuron crosstalk acts through APT2/DHHC7 mediated C3aR-STAT3 activation and synaptic pruning

In conclusion, our study demonstrated that astrocyte IL-1R/NF-κB/C3 signaling mediated the C3 production accelerated microglial APT2/DHHC7 palmitoylation-mediated STAT3 phosphorylation and synaptic pruning (Fig. 10). These findings provide new insights into the pathophysiology of depression that the complement C3 system plays a crucial role in modulating synapses and behaviors. Our finding also suggests that targeting complement systems may provide a new therapeutic approach for treating complement-associated psychiatry diseases at the morphology of synapses.

## Methods

### Animals

C57BL/6 mice (22 ± 2 g; Male, 6 or 8 weeks old) were purchased from the Shanghai Slac Laboratory Animal Center (Shanghai, China). Every four or five mice were placed into one cage (320 × 180 × 160 mm) during a standard 12 h/12 h light (07:00 am on) to dark (07:00 pm off) schedule. The animals have free access to standard chow and water except during behavioral tests. Ambient temperature and relative humidity were maintained at 22 ± 2 °C and 55 ± 5%, respectively. Animals were acclimated the experimental housing conditions for one week prior to the formal experiment. All animal experiments were conducted according to the ethical policies of Huaqiao University and the Chinese Government. The animal procedures were approved by the ethics committee of Huaqiao University (A2019018).

### Reagents

C3aR antagonist SB290157 (M8470) and IL-1R antagonist anakinra (M10139) were purchased by AbMole (Houston, USA). Lipopolysaccharide (LPS, *Escherichia coli* 055:B5, L2880) was purchased from Sigma (St. Louis, USA). Commercial ELISA kits including interleukin-1 (IL-1β, EK0417), interleukin-6 (IL-6, EK0411), and tumor necrosis factor -α (TNF-α, EK0527) were purchased from Boster (Wuhan, China). The primary antibodies used in the present study are shown in Additional file 1: Table S1.

### Drug administration

To demonstrate the proteomic change, mice were randomly divided into the following three groups, respectively: the Control group, the CUMS group, and the CUMS-SB290157 (1 mg/kg) group. Sucrose preference test was conducted at the end of 4-week CUMS.

To test the association of C3/C3aR activation in inflammation-induced depression, mice was injected with LPS (0.83 mg/kg, i.p.), then the behavioral tests were performed 6 h or 24 h later, respectively.

To test the effects of chronic stress on the dynamic C3/C3aR signaling in the prefrontal cortex, depressive-like behaviors and C3/C3aR activation were evaluated once a week during a total 4-week CUMS procedure.

To test whether C3aR blockade restored depression-like symptoms, mice were randomly divided into the following four groups, respectively: the Control/CUMS-vehicle group and the Control/CUMS-SB290157 (1 mg/kg). Animals were intraperitoneally injected with saline, or SB290157, once a day for 4 weeks. The route and dose of SB290157 were based on a recent experimental study showing that SB290157 at 1 mg/kg attenuated neuroinflammation in mice [48].

To test whether C3 was regulated by cytokines, anakinra, an IL-1R antagonist, was treated. Mice were divided into the Control/CUMS-vehicle group, and the Control/CUMS-anakinra (10 mg/kg). Animals were intraperitoneally injected with saline, anakinra once a day for four weeks at a volume of 10 ml/kg body weight. The route and dose of anakinra were based on the recent clinal and experimental studies showing that anakinra at 10 mg/kg attenuated aseptic meningitis in children and ameliorated noise-induced hearing loss in mice [49, 50].

### CUMS

The CUMS procedure was performed for 4 or 8 continuous weeks [51]. Briefly, mild stressors included deprivation of food and water, soiled cage exposure, cage tilt at 45°, exposure to a bottle without water, illumination overnight, space reduction, predator sounds, and light/dark succession. All stressors were randomly scheduled during the entire experiment. The control animals were housed in a separate room without contact with stressors.

### Open-field test

A plastic box (40 cm × 40 cm × 30 cm) divided into 25 equal squares (8 cm × 8 cm) floor was used. Crossing number (crossing with all paws) and rearing number (standing on the hind legs) were recorded in a 5-min session. The number of crossing and rearing were counted by a blinded observer to the stress or the treatment.

### Sucrose preference test

After the open-field test, sucrose preference test was conducted according to a previous publication [52]. Briefly, mice were first trained to drink 1% sucrose solution before the test. Then, food and water were deprived for 12 h. Subsequently, single mouse was placed in a separate cage and had free access to sucrose solution and water for 24 h. Sucrose preference was calculated according to the weight difference before and after the test: Sucrose preference = Sucrose consumption/(Sucrose and water consumption) × 100%.

### Forced swimming test

The detailed procedure used was according to the Porsolt’s method with minor modifications [53]. Briefly, A glass cylinder (25 × 12 × 25 cm) filled with 15 cm high water (23 ± 2 oC) was used as an apparatus. Mice were placed into the cylinder individually for 6 min swimming. The immobility time was recorded only during the final 4 min. The behavior of floating in the water without struggling was considered immobility. The immobility time was counted by a blinded observer to the stress or the treatment.

### Proteomics analysis

The prefrontal cortex tissues from every three mice in each group were dissected and pooled into one biological replicate. The tissues were homogenized with lysis buffer containing protease inhibitors, followed by centrifuging at 16,000*g* for 30 min. The supernatant was retained and used for protein determination by BCA method. The quality of the supernatant was then verified by SDS-PAGE. Subsequently, 100 μg of protein from each sample were incorporated into TEAB buffer, followed by reaction with TCEP, IAM and Trypsin, respectively. Then, the samples were mixed and incubated with TMT (Thermofisher, A44522) for 2 h. The TMT labeled samples were reconstituted with UPLC loading buffer and separated by high-pH liquid with a reversed-phase C18 column. A total of 20 fractions were collected and then combined into 10 fractions. After concentration by vacuum centrifugation, the fractions were dissolved in mass spectrometry loading buffer for second-dimensional analysis by liquid phase tandem mass spectrometry. Protein identifications were performed using the Proteome Discoverer™ Software 2.4.

### Protein extraction procedure and western blot for proteins

After the forced swimming test, the animals were sacrificed. The prefrontal cortex was dissected on an ice plate and was then powdered in liquid nitrogen into several aliquots at − 80 °C.

The tissues were firstly homogenized with RIPA lysate, and the solutions were incubated on ice plate for 30 min. After centrifuging at 12,000*g* for 5 min, the supernatant was taken to measure the protein concentration. The equal protein solution was subjected to SDS-PAGE electrophoresis and transferred to the PVDF membrane. The membrane was blocked with 5% BSA for 1 h followed by the primary antibody incubating overnight. On the secondary day, the membrane was incubated with secondary antibody linked to HRP for 1 h. Finally, ECL solution was added to the PVDF membrane, and the membrane was exposed by Chemiluminescence Imaging System.

### qPCR analysis for proinflammatory cytokines

Total RNA was isolated from the frozen prefrontal cortex with Trizol reagent. RNA concentration was measured by a Micro Analyzer, followed by reversely transcript. The obtained cDNA was subjected to amplification with the primers of IL-1β, IL-6, TNF-α, and internal control GAPDH in which SYBR Green I was used as the signal fluorescence (Additional file 1: Table S2).

### Brain extraction and immunofluorescence

Mice were anesthetized and perfused with ice-cold 0.9% saline and 4% paraformaldehyde, respectively. The brains were removed and postfixed with 4% paraformaldehyde for 24 h. OCT was used to embed the brains after incubation in sucrose solution. Then 15-μm-thick sections were obtained by Leica CM1850 Cryostat and incubated in 4% paraformaldehyde again. The sections were processed by antigen retrieval followed by blocking for 1 h. Subsequently, the sections were incubated with the mixed primary antibodies at 4 °C overnight (Additional file 1: Table S1). Secondary antibodies were added and incubated for 3 h. Then the sections were treated with DAPI for 5 min and observed under confocal microscope (Leica TCS SP8).

### Analysis of proteins expression in single-cell from confocal images

During the immunofluorescence process, the identical settings in confocal microscope were used to acquire images for all samples. The images were photographed, selected, and analyzed from both hemispheres of each animal by a blinded observer to the treatment.

To determine C3/C3aR levels, 20 × plus zoom in single-plane images were collected. Areas of C3/C3aR surface were automatically calculated, and the ratio was used for data analysis.

To determine pSTAT3/APT2 and STAT3/DHHC7 in Iba1 positive cells, 20 × plus zoom in single-plane images were collected. Areas of Iba1 positive surface and protein signals within the surface were automatically calculated, and the ratio was used for data analysis.

To determine C3 colocalized with synaptophysin/PSD95, 20 × plus zoom in single-plane images were collected and analyzed by shortest distance. Ratios of C3 colocalized with synaptophysin/PSD95 were used for analysis.

To determine synaptophysin/PSD95 engulfed in Iba1 positive cells, images with 20 × plus zoom in z stack steps were collected. The 3D morphology of microglia was reconstructed and analyzed. Area and volume ratios of synaptophysin/PSD95 engulfed in microglia were used for data analysis.

### ELISA analysis for proinflammatory cytokines

Commercial ELISA kits were used to measure the concentrations of IL-1β, IL-6, and TNF-α in the prefrontal cortex according to the instruction of the manufacturer.

### Golgi staining

The whole brains were incubated in Golgi-Cox solution for two weeks and then transferred to 30% sucrose solution. Coronal sections were cut into 50 μm thickness. The sections were treated with ammonium hydroxide, rinsed with distilled water, dehydrated and mounted in a resinous medium, respectively. The protrusions in direct continuity with the dendritic shaft were considered spines. The numbers of branch from the dendritic trees were counted.

### Statistical analyses

All data were expressed as mean ± SEM. Normal distribution was firstly confirmed by Kolmogorov–Smirnov test. The proteomic data were analyzed by one-way ANOVA. The data except proteomic analysis were analyzed by two-way ANOVA followed by a Tukey’s test.

## Supplementary Information

**Additional file 1: Table S1.** Primary antibodies information. **Table S2.** Primers used in PCR. **Figure S1.** C3aR is expressed dominantly in microglia, lowly in neurons, but not in astrocytes.

## Data Availability

All data generated or analysed during this study are included in this published article and its supplementary information files.

## Abbreviations

IL-1β: Interleukin-1
IL-6: Interleukin-6
TNF-α: Tumor necrosis factor-α
CUMS: Chronic unpredictable mild stress
