# Supplementary material for: IL-1R/C3aR signaling regulates synaptic pruning in the prefrontal cortex of depression
Source: Cell Biosci. 2022 Jun 17;12:90. doi: 10.1186/s13578-022-00832-4 (PMC9205119; doi:10.1186/s13578-022-00832-4)
Supplement: Supplementary file 1 — Additional file 1: Table S1. Primary antibodies information. Table S2. Primers used in PCR. Figure S1. C3aR is expressed dominantly in microglia, lowly in neurons, but not in astrocytes. [file 13578_2022_832_MOESM1_ESM.pdf]

Supplementary file

IL-1R/C3aR signaling regulates synaptic pruning in the prefrontal cortex of depression

Man-Man Zhang[1], Min-Xia Guo[2], Qiu-Ping Zhang[3], Xue-Qin Chen[4], Na-Zhi Li[2], Qing Liu[1], Jie Cheng[1], Shi-Le Wang[1], Guang-Hui Xu[5], Cheng-Fu Li[3], Ji-Xiao Zhu[2,**], Li-Tao Yi[1,*]

[1]*Department of Chemical and Pharmaceutical Engineering, College of Chemical Engineering, Huaqiao University, Xiamen 361021, Fujian province, PR China*

[2]*Research Center of Natural Resources of Chinese Medicinal Materials and Ethnic Medicine, Jiangxi University of Chinese Medicine, Nanchang 330004, Jiangxi province, PR China*

[3]*Xiamen Hospital of Traditional Chinese Medicine, Xiamen 361009, Fujian province, PR China*

[4]*The First Affiliated Hospital of Xiamen University, Xiamen 361003, Fujian province, PR China*

[5]*Xiamen Medicine Research Institute, Xiamen 361008, Fujian province, PR China*

Corresponding authors:

[*]Li-Tao Yi. Tel./Fax.: 86-592-6162300; *E-mail address*: litaoyi@hqu.edu.cn ORCID: 0000-0001-9052-8607

**Ji-Xiao Zhu. Tel/Fax: 86- 791-87119065; *E-mail address*: zhujx81@sina.com

Running title: IL-1R/C3aR mediates synaptic pruning in depression

Table 1 Primary antibodies information

Antibody	Host	Company	Lot Number	Application	Dilution
Iba1	Goat	Abcam	ab5076	IF	1:200
C3	Rabbit	Abcam	ab97462	IF<br>WB	1:150(IF);<br>1:1000 (WB)
C3aR	Mouse	Santa Cruz	sc133172	IF	1:150(IF);<br>1:1000 (WB)
PSD95	Rabbit	Abcam	ab18258	IF<br>WB	1:100(IF);<br>1:1000 (WB)
PSD95	Mouse	CST	36233S	IF	1:100
Synaptophysin	Rabbit	CST	36406	IF<br>WB	1:100(IF);<br>1:1000 (WB)
Synaptophysin	Mouse	Santa Cruz	sc17750	IF	1:100
pSTAT3	Rabbit	Abcam	ab76315	IF<br>WB	1:100(IF);<br>1:1000 (WB)
STAT3	Mouse	Abcam	9139S	IF<br>WB	1:100(IF);<br>1:1000 (WB)
APT2	Mouse	Santa Cruz	sc515061	IF<br>WB	1:100(IF);<br>1:1000 (WB)
DHHC7	Rabbit	ThermoFisher	PA5-63760	IF<br>WB	1:100(IF);<br>1:1000 (WB)
IL-1R1	Mouse	Santa Cruz	sc-393998	IF<br>WB	1:100(IF);<br>1:1000 (WB)
p-NF-κB p65	Rabbit	Abcam	ab86299	IF<br>WB	1:100(IF);<br>1:1000 (WB)
NF-κB p65	Rabbit	Abcam	ab16502	WB	1:1000
GFAP	Mouse	Santa Cruz	sc-33673	IF	1:150
GFAP	Rabbit	Abcam	ab207165	IF	1:200
NeuN	Rat	Abcam	ab279297	IF	1:200
β-actin	Rabbit	Sigma	A3854	WB	1:5000

Table S2 Primers used in PCR

Gene	Forward	Reverse
IL-1β	5'-TGCCACCTTTTGACAGTGATG-3'	5'-TGATGTGCTGCTGCGAGATT-3'
IL-6	5'-CCCCAATTTCCAATGCTCTCC-3'	5'-CGCACTAGGTTTGCCGAGTA-3'
TNF-α	5'-GATCGGTCCCCAAAGGGATG-3'	5'-CCACTTGGTGGTTTGTGAGTG-3'
GAPDH	5'-TGAGGCCGGTGCTGAGTATGT-3'	5'-CAGTCTTCTGGGTGGCAGTGAT-3'

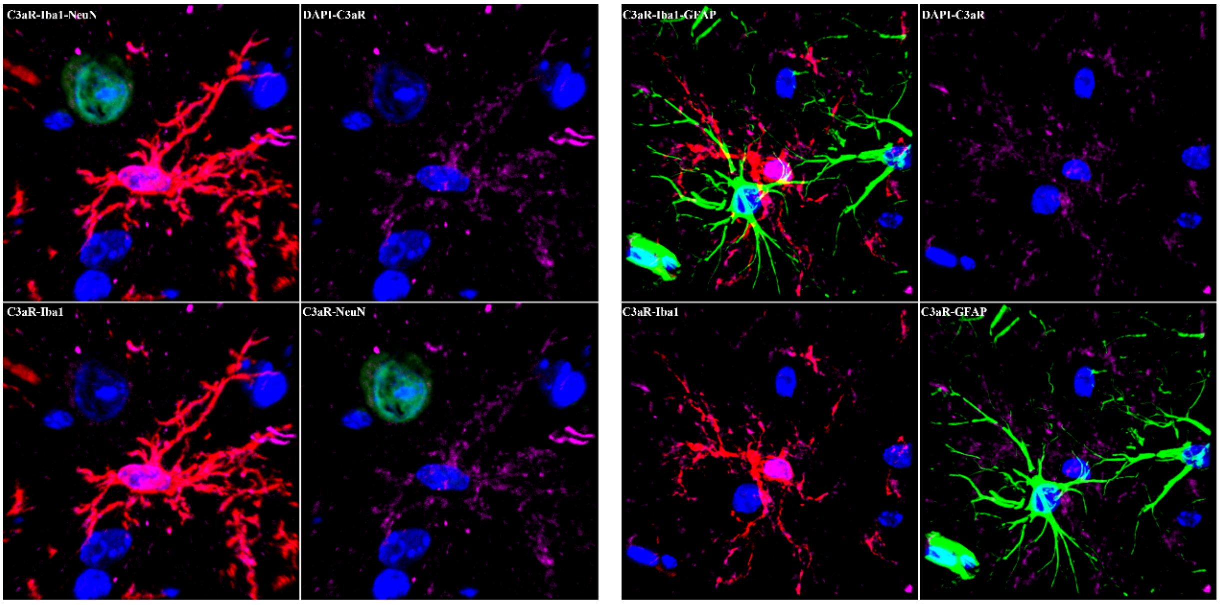

Figure S1 C3aR is highly expressed in microglia, lowly expressed in neuron, but not expressed in astrocyte
